# Supplementary material for: WordCommentsAnalyzer: A windows software tool for qualitative research
Source: F1000Res. 2018 Sep 4;7:536. Originally published 2018 May 3. [Version 2] doi: 10.12688/f1000research.14819.2 (PMC6137411; doi:10.12688/f1000research.14819.2)
Supplement: Supplementary file 3 [file f1000research-7-17614-s0002.tgz › b2a79496-4ada-47c4-9682-a99ce867836d.zip!/Eight Iranian Health Journals/Journal of Research in Rehabilitation Sciences _43.docx]

© Journal of Research in Rehabilitation Sciences . This work is licensed under CC BY-NC 4.0 (https://creativecommons.org/licenses/by-nc/4.0/).

Volume & Issue: دوره 7، شماره 3: 1390

Title: مقایسه دو روش درمان دستی و تیپینگ در بیماران مبتلا به استئوآرتریت زانو

http://jrrs.mui.ac.ir/index.php/jrrs/article/view/244

مقدمه: استئوآرتریت مفصل زانو یکی از شایع‌ترین بیماری‌های تخریبی به خصوص در افراد کهن‌سال است، که منجر به درد و ناتوانی قابل ملاحظه‌ای می‌شود. روش‌های مختلفی برای درمان استئوآرتریت پیشنهاد می‌شود. در مطالعه حاضر از بین درمان‌های ارایه شده دو روش درمان دستی و تیپینگ با هم مقایسه شده است. مواد و روش‌ها: این تحقیق بر روی 36 بیمار میان‌سال (65/6 ± 31/50) مبتلا به استئوآرتریت زانو به روش نمونه‌گیری آسان انجام شد. کلیه بیماران بیش از یک سال درد زانو داشتند. بیماران به صورت تصادفی به دو گروه تقسیم شدند. گروه 1 درمان تیپینگ و گروه 2 درمان دستی. هر گروه قبل از درمان‌های مورد نظر، فیزیوتراپی روتین شامل اولتراسوند و تنس دریافت کردند. درمان در گروه تیپینگ در 6 جلسه و درمان دستی در 10 جلسه هر دو به مدت دو هفته انجام شد. درد بر اساس مقیاس Numerical rating scale (NRS) و سطح توانایی عملکردی بیمار با استفاده از پرسش‌نامه Western ontario mc master universities osteoarthritis (WOMAC) قبل و بلافاصله بعد از درمان ارزیابی شد. (آزمون Paired t-tests جهت مقایسه قبل و بعد از درمان و آزمون Independent t-test جهت مقایسه دو گروه درمانی مورد استفاده قرار گرفت). یافته‌ها: تفاوت معنی‌داری در کلیه متغیرها (به جز خشکی مفصلی) پس از انجام دو روش درمانی مشاهده شد (001/0 > P). در بررسی خشکی مفصلی در گروه تیپینگ قبل و بعد از درمان اختلاف معنی‌داری وجود نداشت (323/0 = P). مقایسه نتایج دو گروه نشان داد که درمان‌های دستی در کاهش علایم استئوآرتریت مؤثرتر بوده است (05/0 > P). نتیجه‎گیری: هر دو روش درمان دستی و تیپینگ در بهبود بیماران استئوآرتریت مؤثر بودند، اما درمان دستی نسبت به تیپینگ روش بهتری جهت کاهش درد و ناتوانی بیماران بوده است. درمان دستی روشی غیر تهاجمی، بدون عوارض و ارزان جهت درمان محافظه کارانه ساییدگی زانو می‌باشد.

Title: بررسی مقایسه ای سن دریافت خدمات درمانی مختلف در بیماران مراجعه کننده به تیم شکاف کام اصفهان

http://jrrs.mui.ac.ir/index.php/jrrs/article/view/272

مقدمه: به علت مشكلات متعدد بیماران مبتلا به انواع شکاف کام و لب، مدیریت درماني آن‌ها كار تیمی و میان رشته‌ای را می‌طلبد. مطالعه حاضر با هدف، بررسي مقایسه‌ای سن شروع گفتار درمانی، جراحی ثانویه کام، پیوند استخوان آلوئول و ارتودنسی در دو گروه مراجعان زودهنگام و مراجعان ديرهنگام به تیم شکاف کام صورت پذيرفت. روش‌ها: در این بررسی 260  پرونده از بیماران مبتلا به انواع شکاف‌های کام و لب بر اساس سن ورود به تيم شکاف کام به دو گروه مراجعان زودهنگام و مراجعان ديرهنگام تقسيم شدند و از نظر چهار متغير ميانگين سن شروع گفتار درماني، ميانگين سن جراحی‌های ثانویه و جراحی آلوئول و سن شروع خدمات ارتودنسی از طریق آزمون T-Test مورد بررسي و مقايسه قرار گرفتند. یافته‌ها: بر اساس نتايج حاصل از اين پژوهش، از ميان 134 نفري كه به گفتار درماني مراجعه كرده بودند، میانگین سن شروع گفتار درمانی در مراجعه كنندگان زودهنگام 3/3 سال و در گروه مراجعه كنندگان ديرهنگام 9 سال، از مجموع 47 نفري كه جراحي ثانويه داشته‌اند، ميانگين سن انجام جراحی در مراجعين زودهنگام 88/3 و در گروه دوم 7/15 سال، از مجموع 17 نفري كه جراحی پيوند استخوان آلوئول داشته‌اند، ميانگين سن انجام جراحی در گروه اول 9 سال و در گروه دوم 69/16 سال و از 24 نفری که خدمات ارتودنسي دريافت كرده‌اند، ميانگين سن شروع ارتودنسي در مراجعين زودهنگام 66/7 و در گروه ديگر 05/17 سال می‌باشد. نتیجه‌گیری: یافته‌های به دست آمده حاکی از آن است که سن انجام جراحی‌های ثانویه و آلوئول و سن شروع خدمات گفتار درمانی و ارتودنسی در مبتلایان به شکاف لب و کام در دو گروه مراجعه كنندگان زودهنگام و ديرهنگام به تيم، تفاوت معنی‌داری دارند.

Title: ارتباط کمر درد و گردن درد با پوسچر بد در زنان شاغل در بیمارستان فاطمه الزهرا نجف آباد

http://jrrs.mui.ac.ir/index.php/jrrs/article/view/274

مقدمه: پیشرفت تکنولوژی و بی‌تحرکی ناشی از آن می‌تواند موجب ایجاد مشکلات پوسچرال شود که آن هم به نوبه خود منجر به بروز اختلالات عضلانی اسکلتی می‌شود. هدف از این مطالعه بررسی ارتباط کمر درد و گردن درد با پوسچر بد در زنان شاغل در بیمارستان فاطمه‌الزهرا نجف‌آباد می‌باشد.  مواد و روش‌ها: در این مطالعه مقطعی توصیفی- تحلیلی تعداد 37 نفر از کارکنان زن بیمارستان فاطمه‌الزهرا نجف آباد با میانگین سنی 36/5 ± 47/32 سال را به صورت تصادفی آسان از بخش‌های مختلف انتخاب نموده و پرسش‌نامه اطلاعات فردی و شرح حال کمر درد و گردن درد توسط فیزیوتراپیست تکمیل گردید، سپس تست‌های مربوط به کمر از آن‌ها به عمل آمده و پوسچر آن‌ها از نماهای قدامی، خلفی و جانبی بررسی شد. اطلاعات تحت نرم‌افزارSPSS با آزمون ارتباط Pearson  تجزیه و تحلیل شد. نتایج: میانگین نمره پوسچر افراد مورد مطالعه 16/1 ±  89/0، میانگین نمره تست کمر آن‌ها 93/1 ± 39/1 و میانگین نمره ریسک ابتلا به اختلالات عضلانی اسکلتی41/6 ± 28/15 بود.شیوع کمر درد و گردن درد در جمعیت مورد مطالعه به ترتیب 4/68 درصد و 9/28 درصد بود. شدت درد به ترتیب با نمره ریسک ابتلا به اختلالات عضلانی اسکلتی، انجام حرکات تکراری، بلند کردن اجسام سنگین، قرار گرفتن در وضعیت‌های بد حین کار و نمره تست کمر ارتباط مستقیم معنی‌داری داشت. بین شدت درد و نمره تست پوسچر ارتباط معنی‌داری یافت نشد. نتیجه‌گیری: نتایج این مطالعه شیوع بالای کمر درد و گردن درد را در زنان شاغل در بیمارستان فاطمه‌الزهرا نجف‌آباد نشان می‌دهد. انجام حرکات تکراری، بلند کردن اجسام سنگین و قرار گرفتن در وضعیت‌های بد حین کار، از مهمترین عوامل مرتبط با کمر درد و گردن درد در جمعیت مورد مطالعه می‌باشند. با توجه به پایین بودن میانگین سنی افراد و بالا بودن میانگین نمره تست کمر (تعداد تست‌های ناموفق کمر) و نمره ریسک ابتلا به بیماری‌های عضلانی اسکلتی، توجه به عوامل ارگونومیک شغلی در جهت کنترل و کاهش اختلالات عضلانی اسکلتی و عوامل خطرزای مربوطه ضروری به نظر می‌رسد.

Title: پايايي و روایی نسخه فارسی آزمون مقياس سنجش فعاليت‌ها دركودكان فلج مغزي (ASK)

http://jrrs.mui.ac.ir/index.php/jrrs/article/view/182

مقدمه: هدف از پژوهش حاضر فارسی‌سازی ابزار سنجش فعالیت‌های روزمره زندگی در کودکان از طریق ترجمه آزمون "مقياس سنجش فعاليت‌ها در كودكان" و بررسی روایی و پايايي نسخه فارسی شده در گروه هدف بود. مواد و روش‌ها: تحقیق حاضر یک مطالعه متدلوژیک است که در آن طی 5 مرحله پرسش‌نامه ASK (Activities scale for kids) به زبان فارسی ترجمه و روایی و پایایی آن بررسی شد. کودکان فلج مغزی از جامعه در دسترس مراجعه کننده به کلینیک‌های کاردرمانی دانشگاه علوم بهزیستی و توان‌بخشی و کودکان سالم به روش گلوله برفی نمونه‌گیری شدند. داده‌های به دست ‌آمده از اطلاعات 73 کودک فلج مغزی و 69 کودک سالم در محدوده سنی 5 تا 15 سال با استفاده از نرم‌افزار SPSS نسخه 13 مورد تجزیه و تحلیل قرار گرفتند و پایایی (تکرار پذیری به فاصله 2 هفته و پایایی درونی) و روایی (محتوا بر اساس روش لاواشه، همزمان با آزمون GMFCS-E and R (Gross motor function classification system-expanded revised) و افتراقی) پرسش‌نامه ASK بررسی شد. یافته‌ها: شاخص روایی محتوای 79/0 برای اساتید و 86/0 برای والدین، روایی محتوای نسخه ترجمه شده آزمون ASK را بالا نشان داد. ضریب همبستگی Spearman بین دو آزمونASK  و GMFCS-E and R 901/0- است که نشان‌ دهنده ارتباط قوی و منفی این دو می‌باشد. Cronbach’ alpha 997/0 نشان دهنده همبستگی درون خوشه‌ای بالای نسخه فارسی آزمون ASK می‌باشد. با استفاده از روش رگرسیون خطی اختلاف معنی‌داری در نمرات آزمون ASK دو گروه کودکان سالم و فلج مغزی مشاهده شد که روایی افتراقی مناسب و بالای این آزمون را نشان می‌دهد. ضریب Pearson 988/0 نشان ‌دهنده همبستگی بالا بین نمرات آزمون ASK در دو بار اجرای آن و پایایی تکرار پذیری قابل قبول این آزمون است. نتیجه‌گیری: نسخه فارسی آزمون ASK از پایایی و روایی قابل قبولی در انواع مورد بررسی برخوردار است و بنابراین می‌تواند ابزار مناسب کلینیک‌ها و همین طور به عنوان یک ابزار تحقیقی به کار رود.

Title: بررسي مقايسه‌اي تأثیر تمرينات Frankel و Swiss Ball بر بهبود بالانس و افسردگي افراد مبتلا به مولتيپل اسكلروزيس

http://jrrs.mui.ac.ir/index.php/jrrs/article/view/278

مقدمه: مولتيپل اسكلروزيس شايع‌ترين بيماري نرولوژي پيش‌رونده در جوانان بالغ مي‌باشد. اين بيماري باعث تخريب ميلين در آكسون‌هاي عصبي شده، در نتيجه ضايعات عملكردي ايجاد مي‌كند. يكي از مناطق مهم درگير در MS مخچه مي‌باشد. مشكل در اين ساختار باعث ايجاد اختلال در تعادل و ... مي‌شود كه به تبع آن كيفيت زندگي كاهش مي‌يابد. بنابراين استفاده از روش‌هاي كم هزينه و بدون عوارض جانبي براي برطرف كردن اين مشكل ضروري به نظر مي‌رسد. مواد و روش‌ها: اين مطالعه از نوع آينده‌نگر، شبه تجربي و يك سويه كور بوده، كه در سال 88-87 در شهر اصفهان انجام گرفته است. در اين مطالعه طي 10 جلسه، به 23 بيمار مبتلا به MS كه در هر گروه درماني قرار مي‌گرفت، تمرينات Frankel يا Swiss Ball به مدت 45 دقيقه در هر جلسه تحت نظارت مستقيم تراپيست آموزش داده مي‌شد. قبل و بعد از كامل شدن جلسات درماني جهت ارزيابي بالانس و افسردگي به ترتيب از پرسش‌نامه‌هاي معتبر Berg balance scale and beck استفاده شد. جهت تجزيه وتحليل داده‌ها با توجه به كمي بودن آن‌ها در هر گروه از آزمون Dependent t-test و بين دو گروه از Independent t-test موجود در نرم‌افزار آماري 13SPSS استفاده شد. یافته‌ها: بين وضعيت تعادل بيماران، قبل و بعد از درمان در گروه تمرينات Frankel و در گروه تمرينات Swiss Ball تفاوت معني‌داري وجود داشت (01/0 > P). بين وضعيت افسردگي بيماران، قبل و بعد از درمان در گروه تمرينات Frankel و در گروه تمرينات Swiss Ball تفاوت معني‌داري وجود داشت (01/0 > P). بين وضعيت تعادل بيماران، قبل از درمان بين دو گروه تفاوت معني‌داري وجود نداشت (18/0 = P). اما بعد از درمان بين دو گروه تفاوت معني‌داري وجود داشت (01/0 > P). بين وضعيت افسردگي بيماران، قبل از درمان بين دو گروه تفاوت معني‌داري وجود نداشت (29/0 = P). همچنين بعد از درمان بين دو گروه تفاوت معني‌داري وجود داشت (30/0 = P). نتیجه‎گیری: انجام تمرينات Frankel و Swiss Ball به صورت مداوم و با فرض ثابت بودن شرايط بيمار باعث بهبودي در تعادل و افسردگي بيماران مبتلا به MS شد. همچنين تمرينات Frankel در بهبود تعادل مؤثرتر از تمرينات Swiss Ball است.

Title: مقایسه تأثیر تقويت کننده‌های پته ای و اجتماعی بر پیشرفت تحصیلی در درس علوم تجربی دانش آموزان دخترکم توان ذهنی

http://jrrs.mui.ac.ir/index.php/jrrs/article/view/180

مقدمه: مطالعات گسترده ای در مورد اثر بخشی تقویت کننده ها بر تغییر رفتار دانش آموزان انجام شده است. اما درباره میزان اثر بخشی تقویت کننده های پته ای بر پیشرفت تحصیلی دانش آموزان کم توان ذهنی تحقیق های کمی وجود دارد. با توجه به این که استفاده از تقویت کننده ها باعث علاقه و انگیزه دانش آموزان به یادگیری می شود، لزوم برنامه ریزی در اجرا و نحوه به کارگیری آن ها اهمیت ویژه ای دارد. هدف از پژوهش حاضر، مقایسه تأثیر تقویت کننده های پته ای و اجتماعی بر پیشرفت تحصیلی دانش آموزان دختر کم توان ذهنی در درس علوم تجربی بود. مواد و روش ها: پژوهش حاضر، یک مطالعه آزمایشی با طرح پیش آزمون- پس آزمون و گروه کنترل بود. 45 دانش آموز دختر پایه سوم راهنمایی کم توان ذهنی در این پژوهش شرکت داشتند که به روش خوشه ای چند مرحله ای از مدارس استثنایی شهرستان های تهران انتخاب شدند. این دانش آموزان به صورت تصادفی به سه گروه 15 نفری تقسیم شدند (دو گروه آزمایش و یک گروه کنترل). برای ارزیابی میزان هوش گروه های مورد مطالعه از آزمون هوشی وکسلر و برای ارزیابی میزان پیشرفت تحصیلی دانش‌آموزان از آزمون معلم ساخته در درس علوم تجربی استفاده شد. اطلاعات جمع آوری شده با استفاده از تحلیل واریانس یک طرفه و آزمون تعقیبی شفه، در نرم افزار SPSS تحلیل شد. یافته ها: میانگین پس آزمون گروه های تقویت پته ای 5/17 (87/0 = SD)، تقویت اجتماعی 48/14 (13/1 = SD) و کنترل 23/13 (23/1 = SD) بود. اختلاف میانگین پيشرفت تحصيلي بین گروه های مورد مطالعه معنی دار بود (001/0P و 03/57 = >(42و2)F). آزمون تعقیبی شفه نشان داد که اختلاف ميانگين گروه تقویت پته ای به طور معنی داري بيشتر از گروه تقويت اجتماعي (001/0P و 02/3) و گروه کنترل(001/0P و 10/4) است. همچنين اختلاف ميانگين گروه تقويت اجتماعي به طور معنی داري بيشتر از گروه کنترل بود (04/0P ; ;و 08/1). نتیجه گیری: با توجه به مطالعه حاضر، تقویت کننده های پته ای در مقایسه با تقویت کننده های اجتماعی و کنترل در پیشرفت تحصیلی دانش آموزان دختر کم توان ذهنی کارایی بیشتری داشتند. همچنین اثربخشی تقویت کننده های اجتماعی در پیشرفت تحصیلی این دانش آموزان بیشتر از گروه کنترل بود. بنابراین، تقویت کننده های پته ای یکی از بهترین برنامه های تقویتی برای دانش آموزان کم توان ذهنی هستند.

Title: مقايسه ويژگي‌هاي الكتروآنسفالوگرام (EEG) در افراد بدون لکنت با افراد مبتلا به لكنت

http://jrrs.mui.ac.ir/index.php/jrrs/article/view/372

مقدمه: علت‌شناسی لکنت و تفاوت‌های موجود میان افراد مبتلا به لکنت و افراد بدون لکنت همیشه موضوعی جالب و در عین حال پیچیده بوده است. شيوع اين اختلال در كل جمعيت حدود 1 درصد تخمين زده شده است. هدف از این مطالعه بررسی تفاوت الگوی Electro-encephalography (EEG) افراد دارای لکنت و فاقد لکنت در شهر اصفهان بوده است. مواد و روش‌ها: این مطالعه تحلیلی و به شیوه مورد- شاهدی دو سو کور روی 31 فرد دارای لکنت و 31 فرد فاقد لکنت که از جنبه‌های سن، جنس و تحصیلات همسان‌سازی شده بودند، انجام شده است. همچنين همه نمونه‌ها از نظر عدم ابتلای به تندگویی (كلاترينگ)، عقب‌ماندگي ذهني، كم‌شنوايي، اختلالات حركتي گفتار و ضايعات مركزي اعصاب و مصرف دارو كه منجر به اختلالات گفتار و زبان می‌شود، بررسی شدند. از تمام نمونه‌ها در شرایط یکسان EEG گرفته شد و نتایج با استفاده از نرم‌افزار SPSS10 و آزمون‌های Fisher's exact test و Independent t-test مورد تجزیه و تحلیل قرار گرفت. یافته‌ها: در مورد متغیرهای فرکانس، دامنه و امواج پاتولوژیک بین دو گروه مورد مطالعه، فرکانس و دامنه تفاوت معنی‌دار نشان دادند ولی در مورد امواج پاتولوژیک اختلاف وجود داشت که این اختلاف معنی‌دار نبود. نتیجه‎گیری: به نظر می‌رسد که تفاوت‌های موجود بین دو گروه مورد مطالعه نشانگر وجود تفاوت در عملکردهای مغزی آن‌ها است و این تفاوت‌ها در پردازش، کنترل، هدایت و اجرای گفتار تأثیر‌گذار است.

Title: شيوع كمردرد و رابطه آن با ميزان فعاليت بدني، سن و شاخص توده بدنی در کارکنان دانشگاه پيام نور استان فارس

http://jrrs.mui.ac.ir/index.php/jrrs/article/view/165

مقدمه: هدف تحقيق حاضر تعيين ميزان شيوع كمردرد و بررسي رابطه آن با ميزان فعاليت بدني، شاخص توده بدنی و سن در کارکنان دانشگاه پيام نور استان فارس بود که با حمايت مالي دانشگاه پيام نور در قالب طرح تحقیقی انجام شده است. مواد و روش‌ها: از ميان 709 نفر از کارکنان دانشگاه پيام نور استان فارس، تعداد 182 نفر زن و مرد شاغل در 12 مرکز به صورت هدفمند و در دسترس (غیر احتمالی) به عنوان نمونه آماري انتخاب شدند. براي ارزيابي کمردرد از پرسش‌نامه استاندارد كيوبك استفاده شد. براي تجزيه و تحليل داده‌ها از آزمون‌هاي آماري 2χ، ضريب همبستگي Pearson و آناليز واريانس يک طرفه (ANOVA) استفاده شد (05/0 < P). یافته‌ها: شيوع بالاي کمردرد (3/86 درصد) در بین نمونه‌های تحقیق و در دامنه سني بين 30 تا 40 سال بود. آزمون 2χ تفاوت معنی‌داری بین افراد دارای کمردرد و نمونه‌هایی نشان داد که هیچ دردی در ناحیه کمر گزارش نکرده بودند (001/0 > P). همچنين تفاوت معني‌داري بين ميزان کمردرد در دو گروه اساتيد و كارمندان مشاهده نشد (05/0 > P). ضريب همبستگي Pearson نشان داد که رابطه معني‌داري بين فعاليت بدني و درد در ناحيه كمر وجود دارد (02/0 = P)، ولي رابطه معني‌داري بين سن و ميزان درد در ناحيه كمر و همچنين جنس و ميزان کمردرد مشاهده نشد (05/0 > P). شاخص توده بدني در بيشتر افراد طبيعي بود. آزمون آناليز واريانس يك طرفه تفاوت معني‌داري بين وزن و ميزان درد در ناحيه كمر نشان داد (02/0 = P). نتیجه‎گیری: افرادی كه ورزش مي‌كردند، درد كمتري داشتند. با توجه به رابطه مثبت ورزش منظم و درد كمتر، پيشنهاد مي‌شود که جهت پيش‌گيري از كمردرد، كاركنان زماني را در هفته به ورزش كردن منظم اختصاص دهند.

Title: بررسی ارتباط میان شکایات صوتی و ویژگی آکوستیکی فرکانس پایه در معلمان دوره ابتدایی شهر اصفهان

http://jrrs.mui.ac.ir/index.php/jrrs/article/view/375

مقدمه: مطالعات نشان می‌دهد که معلمان به دلایلی از جمله استفاده نادرست از صوت، بیشتر از سایرین در خطر ابتلا به اختلالات صوتی هستند. بنابراین بیشتر نیازمند برنامه‌های پیش‌گیری و درمانی می‌باشند، ولی متأسفانه به دلیل محدود بودن کلینیک‌های گفتار درمانی و آسیب‌شناسان گفتار و زبان، همچنین عدم دسترسی به ابزارهای ارزیابی دستگاهی صوت، امکان ارزیابی جامع و دقیق صوتی معلمان در ‌ایران وجود ندارد. با توجه به اهمیت نقش معلمان دوره ابتدایی در تعلیم و تربیت و تأثیر مستقیم سلامت صوتی معلم بر کیفیت تدریس و یادگیری دانش‌آموزان، لازم است تا از ارزیابی‌های ادراکی به شکل مثمر ثمرتری استفاده شود. بنابراین هدف از این پژوهش، بررسی ارتباط میان شکایت‌های صوتی معلمان و نتایج دستگاهی تجزیه و تحلیل فرکانس پایه بود. مواد و روش‌ها: در این مطالعه توصیفی مقطعی، در ابتدا مرور کاملی بر متون حاضر در این زمینه انجام شد و شکایات صوتی رایج در مورد فرکانس پایه، استخراج گردید. این شکایت‌ها پس از تأیید روایی و پایایی، در قالب یک چک‌لیست در اختیار 95 معلم شاغل در مقطع ابتدایی قرار داده شد، که با روش نمونه‌گیری تصادفی- خوشه‌ای انتخاب شده بودند، تا از این طریق صوت خود را ارزیابی نمایند. همچنین نمونه صدای هر معلم نیز ضبط شد تا توسط نرم‌افزار ارزیابی و با نمره‌ای مطابقت داده شود که معلمان برای صوت خود در نظر گرفته‌اند. بدین ترتیب تلقی هر فرد از صوت خود، توسط چک‌لیست و وضعیت فرکانسی، با تجزیه و تحلیل صوتی حاصل آمد. سپس همبستگی این دو نمره محاسبه شد. یافته‌ها: بر اساس مطالعه حاضر همه شکایت‌های صوتی دارای همبستگی معنی‌داری با وضعیت آکوستیکی فرکانس پایه بودند. نتیجه‎گیری: با توجه به معنی‌داری همبستگی تمام شکایات با وضعیت آکوستیکی فرکانس پایه، می‌توان این شکایت‌ها را مبنای قضاوت قرار داد. با این وجود، برای تشخیص اختلال در فرکانس پایه، استفاده از سایر شیوه‌های ارزیابی صوتی نیز لازم به نظر می‌رسد.

Title: رابطه بین ناهنجاری‌های وضعیتی ستون فقرات با عادات و وسایل استراحتی ایرانیان در سنین میان‌سالی در پنج استان منتخب کشور

http://jrrs.mui.ac.ir/index.php/jrrs/article/view/291

مقدمه: از جمله عادات و نیازهای مهم انسان، خواب و استراحت است که بخشی از زندگی افراد را تشکیل می‌دهد و در افراد مختلف به شیوه‌های متفاوت صورت می‌گیرد. هدف از اجراي اين پژوهش، بررسی عادات خوابیدن و وسایل مورد استفاده در جامعه میان‌سال ایرانی، بررسی ارتباط احتمالی بین عادات و شیوه‌های معمول در استراحت و وسایل مورد استفاده هنگام خواب و استراحت است. همچنین مقایسه و شناخت بین تفاوت‌های موجود در شیوه‌های استراحتی ساکنان میان‌سالان در مناطق مختلف ایرانی و به طور کلی روشن‌تر ساختن زمینه‌های ارتباطی بین وسایل استراحتی، شیوه زندگی، وضعیت بدنی و ناهنجاری‌های اسکلتی بود. مواد و روش‌ها: روش انجام اين پژوهش از نوع توصیفی بوده، جامعه آماري آن شامل كليه زنان و مردان میان‌سال (40 تا 55 ساله) استان‌های تهران، یزد، سیستان و بلوچستان، لرستان و کرمانشاه است. با استفاده از روش نمونه‌گيري در دسترس 1719 نفر پرسش‌نامه بررسي نحوه استراحت و وضعيت ستون فقرات را تکمیل کردند. پايايي پرسش‌نامه به روش Cronbach’s alpha 83 درصد برآورد شده است. برای معاینات بالینی حدود 10 درصد از جامعه آماری اولیه، که وجود یکی از ناهنجاری‌های اسکلتی در آن‌ها شایع بوده است (134 آزمودنی)، از استان‌های مختلف به صورت داوطلبانه انتخاب شدند. برای اندازه‌گیری زاویه لوردوز و کیفوز از خط‌کش منعطف و برای تشخیص اسکولیوز از آزمون Adams استفاده شد. داده‌هاي حاصل از پرسش‌نامه‌ها با استفاده از نرم‌افزار آماري SPSS و آزمون‌های آماري t مستقل و LSD برای توصیف تفاوت‌ها در سطح اطمينان 95 درصد مورد استفاده قرار گرفت (05/0 = α). یافته‌ها: بین انتخاب وضعیت خوابیدن به پشت، پهلو و شکم توسط زنان و مردان در استان‌های مختلف تفاوت معنی‌داری وجود داشت (05/0 > P). از نتایج دیگر تحقیق این است که بین استفاده یا عدم استفاده از تشک و بالش توسط زنان و مردان تفاوت معنی‌داری وجود نداشت (05/0 < P)، اما در استان‌های مختلف فقط در مورد استفاده تشک این تفاوت معنی‌دار است (05/0 > P). نتیجه‎گیری: در رابطه با ارتباط بین ناهنجاری‌های اسکلتی و شیوه استراحتی رابطه معنی‌دار آماری بین شیوع لوردوز و خوابیدن روی شکم نشان داده شد. همچنین بین شیوه لوردوز و مواد و نوع بافت وسایل استراحتی رابطه معنی‌دار وجود داشت (05/0 > P). در سایر موارد فرضیه‌های آزمون شده، رابطه معنی‌داری را نشان نداده است.

Title: مقایسه بسامد پایه گفتار و دامنه آن در افراد فارسی زبان ایرانی با سنین متفاوت از هر دو جنس در حین خواندن متن

http://jrrs.mui.ac.ir/index.php/jrrs/article/view/369

مقدمه: در ارزيابي اختلالات صوت به بسامد پایه توجه خاصی می‌شود و به طور معمول انتظار می‌رود که زیر و بمی صوت هر فرد با سن، جنس، و یا شاید جثه، موقعیت اجتماعی، حالت عاطفی، نژاد، زبان و عواملی از این دست متناسب باشد. آگاهی از میزان بسامد پایه در افراد طبیعی می‌تواند معیار مناسبی برای تعیین طبیعی یا غیر طبیعی بودن این ویژگی در افراد دارای اختلال صوت باشد. با توجه به این که در سایر زبان‌ها اطلاعاتی در مورد ویژگی‌های طبیعی صوت در دست است، اما این اطلاعات در مورد زبان فارسی به صورت طبقه‌بندی شده، در سنین متفاوت وجود ندارد. هدف از این مطالعه تعیین میزان بسامد پایه گفتار و دامنه آن در بزرگ‌سالان دارای صوت طبیعی در سنین متفاوت در دو جنس بود. مواد و روش‌ها: پژوهش حاضر، یک مطالعه توصیفی تحلیلی است که به صورت مقطعی انجام شد. جامعه مورد مطالعه در این پژوهش، 200 نفر از بزرگ‌سالان باسواد دارای صوت طبیعی 20 تا 59 ساله بودند. صدای آزمودنی‌ها حین خواندن متن رنگین‌کمان ضبط شد. تجزيه و تحليل آزمايشگاهي نمونه‌هاي صوت با نرم‌افزار Dr.Speech انجام شد و داده‌ها با آزمون t مورد تجزیه و تحلیل قرار گرفت. یافته‌ها: بسامد پایه و دامنه تغییرات آن در زنان در سنین متفاوت به طور معنی‌داری بالاتر از مردان است. نتیجه‎گیری: بسامد پایه و دامنه تغییرات آن در افراد طبیعی با سن و جنس متفاوت، متفاوت است. این تفاوت مربوط به ساز و کار ساختاری و فیزیولوژیکی و سایر موارد است.

Title: بررسی سطوح شناختی بیماران اسکیزوفرنیک بر اساس غربالگری سطح شناختی آلن

http://jrrs.mui.ac.ir/index.php/jrrs/article/view/184

مقدمه: مهارت شناختی فرایندی بسیار پیچیده است و اساس عملکرد هدفمند بشر را رقم می‌زند. تأثیر اختلال شناختی را در عملکرد بیماران روان‌پزشکی و نورولوژیک به وضوح می‌توان دید. بنابراین ارزیابی دقیق از ظرفیت شناختی این افراد به منظور پیش‌بینی عملکرد آن‏ها ضروری است. غربال‌گری سطح شناختی Allen، برآوردی سریع از ظرفیت یادگیری و توانایی‏های باقیمانده بیمار مبتلا به اختلال شناختی ارایه می‌دهد. مواد و روش‌ها: مطالعه به شیوه توصیفی- تحلیلی از سطح شناختی 60 بیمار اسکیزوفرنیک 40-18 سال در دو گروه بستری دایم و سرپایی و 30 نفر از افراد سالم، بر اساس غربال‌گری سطح شناختی Allen صورت گرفت. در گروه بیماران بین سطح شناختی افراد بستری و سرپایی مقایسه‏ای صورت گرفت و اثر متغیرهای دموگرافیک بر سطح شناختی بررسی شد. نتایج با روش‏های آماری ضریب همبستگی Pearson و آنالیز واریانس یک‏طرفه توسط نرم‌افزار 11SPSS بررسی شد. یافته‌ها: نتایج حاکی از معنی‌داری اختلاف میانگین سطح شناختی گروه سالم و بیمار (001/0 > P) و نیز بیماران بستری و سرپایی (001/0 > P) بود. سطح شناختی با سن بروز بیماری (594/0 = r) و سطح تحصیلات (545/0 = r) ارتباط مستقیم و با مدت ابتلا به بیماری (574/0- = r) و مدت اقامت بستری (454/0- = r) ارتباط معکوس داشت. بین متغیرهای دموگرافیک و سطح شناختی افراد سالم ارتباط معنی‌داری وجود نداشت. نتیجه‎گیری: گروه افراد سالم از سطح شناختی بالاتری برخوردار بودند و سطح شناختی بیماران بستری نسبت به بیماران سرپایی پایین‌تر بود. سن بروز بیماری، مدت ابتلا به بیماری، مدت اقامت بستری دایم و سطح تحصیلات بر سطح شناختی بیماران اسکیزوفرنیک مؤثر بودند.

Title: تأثیر جایگاه الکترودی بر پارامترهای P300 شنوایی در افراد هنجار

http://jrrs.mui.ac.ir/index.php/jrrs/article/view/329

مقدمه: به منظور کسب آگاهی از فرایند عملکرد قشری، پتانسیل‌های وابسته به رخداد، بسیار مناسب هستند. از فاکتورهای تأثیرگذار بر P300، جایگاه الکترودی می‌باشد. پژوهش حاضر به منظور مقایسه دامنه و زمان نهفتگی پتانسیل وابسته به رخداد P300 شنوایی در جایگاه‌های الکترودی مختلف در افراد هنجار در محدوده سنی 50-18 سال انجام شده است. مواد و روش‌ها: در اين پژوهش مقطعي و غير مداخله‌اي،P300 شنوایی به روش تحریک متفاوت با استفاده از دو محرک تن برست بر روی 27 فرد هنجار با میانگین سنی 2/23 سال، انجام شد. یافته‌ها توسط آزمون آماری Paired t-test و نرم‌افزار 16SPSS مورد تحلیل قرار گرفت. یافته‌ها: میانگین دامنه و زمان نهفتگی موج P300 در افراد هنجار و در هر دو جنس در جایگاه‌های الکترودی مختلف اختلاف معنی‌داري داشت (05/0 > P). نتیجه‎گیری: محل قرارگیری الکترود فعال در ثبت P300 مؤثر است. برای ثبت P3b یا همان P300، بهتر است از جایگاه خلفی‌تر استفاده شود.

Title: تأثیر برنامه تمرینی 8 هفته‌ای بر سندرم درد پاتلوفمورال

http://jrrs.mui.ac.ir/index.php/jrrs/article/view/154

مقدمه: سندروم درد پاتلوفمورال، یکی از شایع‌ترین مشکلات زانو است، که جوانان و نوجوانان آن را تجربه می‌کنند. هدف از این مطالعه، بررسي تأثیر يك برنامه تمريني 8 هفته‌اي بر روی افراد داراي سندرم درد پاتلوفمورال است. مواد و روش‌ها: جامعه آماري تحقيق حاضر را 290 نفر از دانش‌آموزان پسر 17 تا 19 سال مقطع دبیرستان تشکیل دادند. نمونه‌هاي آماري 60 نفر از دانش‌آموزان مبتلا به سندرم درد پاتلوفمورال بوده، كه به صورت تصادفي در دو گروه تجربي (30 = n) و گروه شاهد (30 = n) تقسيم شدند و در مدت 8 هفته مورد مطالعه قرار گرفتند. گروه تجربي يك برنامه تمريني متشكل از تمرينات كششي و تقويتي را به صورت سه جلسه در هفته انجام داد. شدت درد بر اساس مقياس سنجش عددي و عملكرد حركتي بر اساس مقياس Kujala در ابتدا و در پايان 8 هفته مورد ارزيابي قرار گرفت. در تجزيه و تحليل داده‌ها از نرم‌افزار SPSS13 استفاده شد. یافته‌ها: متغيرهاي سن، قد، وزن، شاخص جرم بدن و مدت زمان وجود درد مورد ارزيابي قرار گرفت و با استفاده از آزمون Mann withney مشخص شد كه بين دو گروه تجربي و شاهد از نظر اين متغيرها اختلاف معني‌داري وجود نداشت. در مقایسه بین دو گروه تجربی و شاهد قبل از انجام 8 هفته تمرین در شاخص شدت درد و عملكرد حركتي اختلاف معني‌داري مشاهده نشد (05/0 < P). در میزان درد در گروه تجربی قبل و بعد از اجرای پروتکل تمرینی، تفاوت معنی‌داری مشاهده شد (05/0 > P). در ميزان عملكرد حركتي در گروه تجربي قبل و بعد از اجراي پروتكل تمريني، تفاوت معني‌داري مشاهده شد (05/0 > P). همچنين در میزان درد بين گروه تجربی و گروه شاهد بعد از اجرای پروتکل تمرینی، تفاوت معنی‌داری مشاهده شد (05/0 > P). در میزان عملکرد حرکتی زانوی افراد بین گروه تجربی و گروه شاهد بعد از اجرای پروتکل تمرینی، تفاوت معنی‌داری مشاهده شد (05/0 > P). نتیجه‎گیری: با توجه به یافته‌های پژوهش حاضر، تمرين درماني مي‌تواند در كاهش درد و بهبود عملكرد حركتي افراد مبتلا به سندرم درد پاتلوفمورال مؤثر باشد.

Title: مقایسه قسمت واج شناختی حافظه فعال در کودکان پیش دبستانی مبتلا به لکنت با کودکان طبیعی

http://jrrs.mui.ac.ir/index.php/jrrs/article/view/243

مقدمه: یکی از حیطه‌های زبانی مرتبط که اخیراً توجه بیشتری به آن در متون مربوط به لکنت شده است، قسمت واج‌شناختی حافظه فعال می‌باشد. هدف مطالعه حاضر مقایسه توانایی‌های قسمت واج‌شناختی حافظه فعال در کودکان طبیعی و کودکان مبتلا به لکنت می‌باشد.  مواد و روش‌ها: 30 کودک 6-4 ساله مبتلا به لکنت با 30 نفر از هم‌سالان طبیعی خود که از نظر سن همتاسازی شده بودند مورد مقایسه قرار گرفتند. از آزمون تکرار فوری ناکلمه برای تعیین زمان واکنش و تأثیر طول ناکلمه بر صحت تولید و روانی کلامی پاسخ‌ها استفاده شد. تکلیف دوم تکلیف تکرار با تأخیر ناکلمه بود که از آن برای سنجش سرعت و کفایت یادگیری نا‌کلمه استفاده شد.  یافته‌ها: آنالیز بین دو گروه تفاوت‌های مشخصی را در همه شاخص‌ها به جز صحت ناکلمه نشان داد. میانگین زمان واکنش در کودکان مبتلا به لکنت طولانی‌تر از گروه کنترل بود و همچنین سرعت یادگیری نا‌کلمه‌ها در کودکان لکنتی کندتر از گروه طبیعی بود و این تفاوت‌ها معنی‌دار بود. به علاوه، تحلیل روانی پاسخ‌های کودکان لکنتی در حین تکرار ناکلمات با طول متفاوت نشان داد که به همان میزان که طول ناکلمه افزایش می‌یابد درصد ناروانی نیز افزایش می‌یابد و این اختلاف معنی‌دار بود. همچنین اگر چه درصد ناکلمات صحیح به طور کلی و در حین افزایش طول ناکلمه در کودکان لکنتی در مقایسه با گروه کنترل کمتر بود، اما این اختلاف معنی‌دار نبود.  نتیجه‌گیری: نتایج مطالعه حاضر، از تحقیقات پیشین که نشان دادند توانایی‌های حافظه فعال واج‌شناختی در کودکان مبتلا به لکنت نسبت به کودکان طبیعی درجاتی تأخیر و کندی دارند، حمایت می‌کند.

Title: رابطه قوس طولی کف پا با برخی شاخص‌هاي حرکتی منتخب کودکان 11 تا 14 ساله

http://jrrs.mui.ac.ir/index.php/jrrs/article/view/209

مقدمه: هر گونه تغییر در ساختار آناتوميكي پا و موقعيت قرارگيري آن در پايين‌ترين بخش زنجيره حركتي اندام تحتاني و سطح اتكاي به نسبت كوچك آن، اجرای مهارت‌های حرکتی را تحت‌ تأثیر قرار می‌دهد. پژوهش حاضر با هدف بررسي، رابطه تغییرات قوس طولی کف پا با برخي شاخص‌هاي حرکتي کودکان 11 تا 14 ساله انجام شد. مواد و روش‌ها: تحقيق حاضر از نوع توصیفی- همبستگی است كه به اين منظور 211 دانش‌آموز دوره راهنمايي به صورت تصادفي انتخاب شدند و با توجه به میزان افت استخوان ناوی در سه گروه کف پاي صاف (58 نفر)، طبيعي (106 نفر) و گود (47 نفر) قرار گرفتند. ابزارهاي اندازه‌گيري شامل جعبه آينه، ثبت نقش کف پا روي زمين و اندازه‌گيري افت استخوان ناوی برای بررسی میزان قوس طولی کف پا و پارامترهای حرکتی با آزمون‌هاي استاندارد بود. داده‌‌ها به وسيله روش‌های آماری ضريب همبستگي رتبه‌اي Spearman، تحليل تشخيص، تحليل واريانس و آزمون تعقيبي Bonferroni در نرم افزار15 SPSS و سطح معني‌داري 05/0 مورد تجزیه و تحلیل قرار گرفتند. يافته‌ها: نتايج تحقيق نشان داد كه به غير از تعادل ارتباط معنی‌داری بین هيچ كدام از پارامترهای توانايي‌هاي حرکتي با ناهنجاری‌های کف پای صاف و گود وجود ندارد. همچنين تفاوت معني‌داري بين گروه‌ها در توانايي‌هاي حركتي به غير از تعادل يافت نشد. بحث: مي‌توان گفت كه رابطه معني‌داري بین توانايي‌ها و مهارت‌هاي حرکتي مورد بررسی در این تحقیق (به غير از تعادل) با میزان قوس طولی کف پا وجود ندارد.

Title: بررسی رشد برخی خوشه‌های همخوانی در کودکان 2 تا 4 ساله فارسی زبان

http://jrrs.mui.ac.ir/index.php/jrrs/article/view/374

مقدمه: خوشه همخوانی، آمدن پیاپی چند همخوان در یک هجا است، بدون آن که واکه‌ای در میان این همخوان‌ها قرار گیرد. آن چه در این مورد حایز اهمیت است، سن اکتساب خوشه‌های همخوانی می‌باشد. توانایی تولید خوشه‌های همخوانی در حدود دو سالگی گزارش شده است. اغلب این تلاش‌ها منجر به ساده‌سازی خوشه‌های همخوانی شده، تا سرانجام خوشه‌های همخوانی به صورت صحیح تولید شود. هدف از پژوهش حاضر، تعیین سن اکتساب و الگوهای رشدی برخی از خوشه‌های همخوانی در کودکان فارسی زبان است. مواد و روش‌ها: پژوهش حاضر یک مطالعه توصیفی است. جامعه مورد مطالعه شامل 120 کودک طبیعی 2 تا 4 ساله فارسی زبان شهر اصفهان بود. در این پژوهش توانایی تولید خوشه‌های همخوانی با استفاده از نامیدن تصاویر بررسی شد. ابزار مورد استفاده محقق ساخت و شامل 24 واژه با ساختار cvcc بود. یافته‌ها: سن اکتساب دو خوشه همخوانی mp و nd، حدود 3-7/2 سالگی، خوشه‌های همخوانی xt، sk، sb، ∫k، st، ft، ∫t، ks، bz، f∫، ng و ∫m حدود 6/3-1/3 سالگی و tr، rx، rd، rg، rf، rm و rs حدود 4-7/3 سالگی بود. نتیجه‎گیری: سن اکتساب خوشه‌های همخوانی از سن 3-7/2 آغاز شد. اگر به ترتیب اکتساب خوشه‌های همخوانی توجه شود، ملاحظه می‌شود که رشد خوشه‌های همخوانی از رشد همخوانی تبعیت می‌کند. خوشه‌های همخوانی که حاوی همخوان‌های انسدادی و خیشومی می‌باشند، قبل از خوشه‌های همخوانی دیگر کسب می‌شوند.

Title: تاثیر 8 هفته تمرینات هوازی بر کیفیت زندگی و درد بیماران مبتلا به آرتریت روماتوئید

http://jrrs.mui.ac.ir/index.php/jrrs/article/view/205

هدف: آرتریت روماتوئید یک بیماری التهابی است که در آن درد مفاصل، مهم‌ترین مشکل افراد مبتلا به این بیماری به حساب می‌آید. درد و خشکی مفاصل باعث تحرک کم این افراد و در نهایت کاهش کیفیت زندگی این بیماران می‌شود. از این رو، هدف از این مطالعه تأثیر تمرین هوازی بر کیفیت زندگی و درد بیماران مبتلا به آرتریت روماتوئید بود. مواد و روش ها: این مطالعه از نوع پیش‌آزمون- پس‌آزمون با گروه شاهد است. در این پژوهش بیماران 65-38 ساله مبتلا به آرتریت روماتوئید بیمارستان الزهرا که تحت درمان بودند و هیچ گونه تغییر دارویی در طول دوره درمان نداشتند، مورد بررسی قرار گرفتند. بیمارانی که سابقه هیچ گونه فعالیت ورزشی در طی شش ماه گذشته را نداشتند و به هیچ بیماری حاد و مزمن یا ذهنی و روانی مبتلا نبودند، به عنوان نمونه‌های تحقیق در نظر گرفته شدند (23 نفر). نمونه‌ها به طور تصادفی به دو گروه شاهد (12 نفر) و آزمون (11 نفر) تقسیم شدند. گروه آزمون به مدت 8 هفته تمرین هوازی کار با دوچرخه کارسنج را انجام دادند و گروه شاهد هیچ گونه فعالیت ورزشی را نداشتند. این مطالعه از نوع نیمه تجربی بود و برای گردآوری داده‌ها از فرم کوتاه 36 برای سنجش کیفیت زندگی و مقیاس دیداری خطی درد برای ارزیابی میزان درد استفاده شد. میزان درد و کیفیت زندگی بیماران قبل و بعد از 8 هفته بررسی شد. در نهایت داده‌ها با استفاده از نرم‌افزار SPSS و آزمون‌های t زوجی و t مستقل مورد تجزیه و تحلیل قرار گرفت. یافته‌ها: اختلاف قابل توجهی میان نمرات کیفیت زندگی، در ابتدا و انتهای مداخله در گروه آزمون وجود داشت (01/0 > P)؛ در صورتی که این تفاوت در گروه شاهد معنی‌دار نبود (05/0 < P). همچنین شدت درد در گروه آزمون (05/0 > P) نسبت به گروه شاهد (05/0 < P) کاهش معنی‌داری را نشان داد. نتیجه‌گیری: تمرینات هوازی می‌تواند شدت درد را در بیماران آرتریت روماتوئید کاهش داده، باعث بهبود کیفیت زندگی این بیماران شود.

Title: بررسی تأثیر آموزش زبان انگلیسی بر میزان شدت لکنت

http://jrrs.mui.ac.ir/index.php/jrrs/article/view/273

مقدمه: هدف از این پژوهش بررسی تفاوت تأثیر آموزش زبان انگلیسی بر میزان شدت انواع لکنت در زبان اول و زبان انگلیسی افراد یک زبانه دارای لکنت بوده است. مواد و روش‌ها: جامعه آماری این پژوهش کلیه افراد 12 تا 23 ساله دارای لکنت بودند، که به مراکز گفتار درمانی شهر اصفهان مراجعه کرده‌اند. نمونه مورد مطالعه 15 نفر بودند که به روش تصادفی ساده انتخاب شدند. روش پژوهش شبه آزمایشی و طرح پژوهش یک طرح تک گروهی پیش‌آزمون– پس‌آزمون است. در این پژوهش آزمودنی‌ها قبل و بعد از آزمایش از نظر میزان شدت لکنت مورد سنجش قرار گرفته‌اند. در این پژوهش میزان شدت انواع لکنت با استفاده از تست SSI3 Howell و همکاران مورد اندازه‌گیری و سنجش قرار گرفت. یافته‌ها: آموزش زبان انگلیسی به روی میزان شدت لکنت تأخیری در کلمات عملکردی و کلمات محتوایی در زبان انگلیسی نسبت به زبان اول تأثیر معنی‌داری ندارد، اما آموزش زبان انگلیسی در کاهش میزان لکنت غیر تأخیری در کلمات عملکردی و کلمات محتوایی در زبان انگلیسی نسبت به زبان اول تأثیر معنی‌داری دارد. همچنین آموزش زبان انگلیسی در کاهش میزان شدت کل لکنت در زبان انگلیسی نسبت به زبان اول تأثیر معنی‌داری دارد. این اثرات حداقل در سطح 05/0 = P معنی‌دار بودند. نتیجه‎گیری: آموزش زبان انگلیسی پس از 12 سالگی تأثیر مثبت و یا منفی در درمان و بهبود لکنت در زبان اول افراد لکنتی نداشت. همچنین پس از مهارت گویش آموزش زبان انگلیسی، میزان شدت لکنت در زبان انگلیسی نسبت به زبان اول کمتر شد.

Title: مروری بر یافته‌های پژوهشی مربوط به کشش استاتیکی بر روی افراد سالم

http://jrrs.mui.ac.ir/index.php/jrrs/article/view/294

كشش يكي از قديمي‌ترين روش‌هاي درماني است كه در بين فرهنگ‌هاي باستاني داراي جايگاه ويژه‌اي بوده است. اين روش در كنار روش‌هاي دستي ديگر شامل ماساژ و جااندازي مفاصل، جزء آموزش‌هاي سنتي طب قديم به شمار مي‌آمده است. امروزه پژوهش‌هاي وسيعي در خصوص تأثيرات اين روش دستي بر روي گروه‌هاي عضلاني مختلف در بيماران و همچنين افراد سالم ورزشكار انجام پذيرفته است. همچنین روش‌های گوناگونی جهت اعمال کشش بر بافت‌های بدن تعریف شده است. یکی از این روش‌ها، کشش ایستا یا استاتیکی است که به ویژه در دهه‌های اخیر مورد توجه پژوهشگران قرار گرفته است. گر چه در مجموع می‌توان از نتایج این پژوهش‌ها پی برد كه کشش استاتیکی اثرات طولانی مدتی بر تحرک مفاصل و كاهش مقاومت بافتی داشته و جهت ماندگاري اثرات كشش بر بافت‌ها، نیاز به صرف زمان حدوداً دو ماهه می‌باشد؛ لیکن هدف از این نوشتار بررسی جزییات مبتنی بر پژوهش‌هایی است که در ارتباط با کشش استاتیکی بر روی افراد سالم طی سه دهه اخیر انجام پذیرفته است. شاید در کنار هم قرار گرفتن برخی از یافته‌های پژوهشی بتواند تصمیم‌گیری در خصوص اعمال کشش بر ماهیچه‌های مختلف را برای درمانگران ساده‌تر سازد.
